# Lessons learned from implementing health systems science and community service course for fourth-year medical students

**DOI:** 10.1186/s12909-025-07137-3

**Published:** 2025-04-15

**Authors:** Sarah B. Siddiqui, Kathleen M. Everling, Premal Patel, Hani Serag

**Affiliations:** 1 Division of General Internal Medicine, Department of Internal Medicine, University of Texas Medical Branch at Galveston, Galveston, TX USA; 2 Office of Educational Development, John Sealy School of Medicine, University of Texas Medical Branch at Galveston, Galveston, TX USA; 3 Division of Infectious Disease, Department of Medicine, Long School of Medicine, The University of Texas at San Antonio, San Antonio, TX USA; 4 Department of Population Health and Health Disparities, School of Public and Population Health, University of Texas Medical Branch at Galveston, Galveston, TX USA

**Keywords:** Health systems science, Community service, Service-learning, Curriculum implementation

## Abstract

**Background:**

With the call to integrate health systems science (HSS) in medical education curriculum comes the need for more practical guidance from implementation experiences. In June 2020, the University of Texas Medical Branch John Sealy School of Medicine implemented a required course entitled “Health Systems Science and Community Service (HSS)” for fourth-year medical students. This quality improvement study describes the course and the lessons learned in the first four iterations.

**Methods:**

The course was formatted as a 10-month-long, asynchronous course that ran concurrently with post-clerkship rotations. Throughout the four iterations, we used close- and open-ended questions to systematically collect students’ feedback twice annually. Descriptive analysis of quantitative data was performed, and general themes were drawn from qualitative items from the evaluations. In addition, the National Board of Medical Examiner (NBME) HSS Subject Examination was administered in the course during the third and fourth iterations; student performance was analyzed. The course contents were iteratively revised each year to incorporate more HSS and community service components.

**Results:**

The course cohorts 1 through 4 from each iteration had 234, 221, 220, and 217 students, respectively. The response rates for the end-of-year course evaluations were 91% for cohorts 1, 2, and 3, and 94% for cohort 4. Most students reported that the amount of material they were required to cover was reasonable across all four iterations. While most respondents from cohorts 1, 2, and 4 found the number of community service hours reasonable, the majority of respondents in cohort 3 indicated it was excessive. The common themes from students’ responses across cohorts included that some of the course content should have been taught in earlier years of their medical training, that community service activities are valuable but should have been optional, and that students appreciated the flexibility of the course. Student performance on the NBME exam was comparable to national performance. We summarized the iterative changes for each cohort based on feedback.

**Conclusions:**

We described the implementation of a post-clerkship course on health systems science integrating with community service and service-learning. A major lesson learned was the need to take an iterative approach in building components like service-learning and addressing challenges, including student buy-in.

**Supplementary Information:**

The online version contains supplementary material available at 10.1186/s12909-025-07137-3.

## Introduction

Rooted in the 1910 Flexner Report commissioned by the Carnegie Foundation on Medical Education in the United States and Canada [1], basic and clinical sciences have represented dominant foundational components of medical education for the last century [2, 3]. Health systems in the United States and worldwide have since witnessed significant changes in terms of policies, technologies, and approaches. Such transformations in health systems have required changes in the education and training of health professionals, including physicians, to ensure they are well-equipped to practice within these systems. The shift toward patient-centered care requires competencies such as demonstrating cultural humility, taking a holistic and systems-based approach, and practicing effective communication and coordination. Additionally, the increased complexity of cost recovery and the significant rise in the use of telemedicine, digital health tools, and electronic medical records have increased the need for additional competencies in health financing, clinical informatics, and health technology [4, 5].

Health systems science (HSS) has evolved to address these competency gaps as a third educational pillar alongside the basic and clinical sciences [3]. HSS provides a unified framework of various domains to contextualize practicing healthcare and caring for patients and populations within an adaptive and dynamic system [6]. Gonzalo et al. suggested that integrating HSS into medical education empowers future physicians to become *systems citizens* who strive to improve patient care and population health [7]. It is no coincidence that this transformation in medical education is occurring at a time when healthcare and public health systems are increasingly fragmented, and there is growing awareness of health inequities and disparities.

Since there is no standardized approach for implementing the HSS curriculum in medical education, we integrated it into the curriculum by aligning it with community service and service-learning, emphasizing their crucial overlapping concepts and skills. There exists rich literature describing the benefits of implementing community service and service-learning into the medical school curriculum, including associations with positive student-centered outcomes like enhanced communication skills, higher empathy levels, better interpersonal relationships (e.g., patient-physician relationships), improved leadership skills, better knowledge of population health, and greater ability to determine community needs [8]. It helps future healthcare professionals identify existing community resources and employ them to address social determinants of health (SDOH) and provide comprehensive patient care [9, 10]. While community service focuses on providing assistance, the value of service-learning lies in its emphasis on the learning process that occurs through the service activity. Service-learning is an integrated part of the course curriculum that allows learners to reflect on their experience and analyze their actions to enhance their understanding. With these positive outcomes in mind, the service-learning approach helps develop value-added roles for medical students. Value-added roles allow for creating learning experiences that add value to health systems, patients, and the community, along with addressing critical HSS-related competencies such as population, public, and SDOH, health systems improvement, and value-based care.

## Methods

### Overview

Accreditation bodies for medical education require medical schools to focus on content and competencies that are systems-level related. The Liaison Committee on Medical Education (LCME), which is the accrediting body for North American allopathic medical schools, requires curricular content that overlaps with HSS domains [11]. For example, LCME Standard 7.1 has behavioral and social sciences listed with the biomedical sciences [11]. Other required LCME curricular content that closely overlaps with HSS includes Standard 7.5 Societal Problems; 7.6 Structural Competence, Cultural Competence and Health Inequities; 7.7 Medical Ethics; and 7.9 Interprofessional Collaborative Skills [11]. In addition, LCME requires that part of competencies include Standard 6.6, in which the medical school “provides sufficient opportunities for, encourages, and supports medical student participation in service-learning and/or community service activities” [11].

A major challenge in undergraduate medical education is how to integrate HSS into an already densely packed curriculum. Despite the growing literature on HSS curriculum implementation [12], there is a need for more studies drawing on implementation experiences for practical guidance on how to approach HSS integration. Some medical schools have adopted longitudinal HSS threads across all years of training, while others have implemented them during a specific phase, like pre-clerkship or clerkship [13].

While the ideal integration of HSS is to implement it as a longitudinal thread across training years, various challenges and barriers (including limited resources, limited time, and/or leadership buy-in) may prevent this approach from being realistically used in the initial phase. The John Sealy School of Medicine (JSSOM) of the University of Texas Medical Branch (UTMB) has a four-year traditional program with a large, diverse student class size. Each class is approximately 236 students, with slightly more females than males and with students identifying as approximately 25% Asian, 20% Hispanic or Latino of any race, 35% White, 10% African American, and 10% identifying in other categories, including non-identified and as two or more races.

In circa 2019, an opportunity was identified to develop an HSS course as the post-clerkship phase was being restructured. We decided to integrate community service and service-learning as essential parts of this required post-clerkship course. We launched the Health Systems Science & Community Service Course in June 2020. When developing and implementing the course, we took on an iterative approach, including rolling out the components of the community service requirements at different stages. In this paper, we describe the course evolution across four years, summarize the results of student feedback and student exam performance, and highlight the lessons learned. Our institutional IRB has classified this study as QI/QA. The JSSOM Educational Research Committee at our institution also approved this study.

The Health Systems Science and Community Service Course had gone through four iterations from June 2020 to April 2024. The overall course goals were for students to increase their knowledge and understanding of the various factors that potentially impact patient care and build skills that enable them to grow into physicians who meaningfully improve the health of patients and populations. The course was mandatory and asynchronous, which fourth-year medical students took concurrently with their other post-clerkship rotations. This was a significant change to the fourth-year curriculum, as this course was one of the first required post-clerkship courses. Previously, fourth-year students were only required to take electives, including Acting Internships and Selectives. Selectives were required courses that may have multiple options grouped by themes such as medical humanities, so there was an element of choice within the requirement, but the Selective itself was a required course.

Recognizing that the fourth year was a critical year for medical students who take Acting Internships, away rotations, and Step 2 examinations, along with applying and interviewing for residency, we identified the need for flexibility within the curriculum. We formatted the course into a 10-month course that ran from June to April and focused on developing the curriculum from the conceptual framework of experiential learning. Using an iterative, systematic approach is well-established in educational literature. Briggs et al. (2003) described the continuous and frequent planning criterion for continuous quality improvement in academic planning and the need for the “use of evaluation for adaptive change.” [14] The evaluation process included plans to apply lessons learned to the curriculum, “making use of results to improve learning outcomes or the learning experience. Thus, the evaluation process often repeats as educators apply lessons learned and then evaluate and iterate the improved curriculum.” [15] In each iteration, we made changes based on implementation experience, feedback from students, the course committee, and the curriculum committee, along with educational resource availability.

The course structure included community service, quarterly assignments, and assessments. Each component evolved across the four iterations. Initially, the community service component consisted of community service; later, service-learning was added. It is worth differentiating between community service and service-learning as adopted in our course. Community service and service-learning both benefit medical education but differ in purpose and design. Community service is typically volunteer-based that aims at benefiting the community, often without direct ties to academic coursework, while service-learning integrates community work with curriculum goals, reflection, and mutual benefit. Educational studies consistently show that service-learning more effectively builds empathy and professional growth in medical students [16, 17]. 

For the completion of community service hours, in most iterations, we allowed students to choose where they engage with community service, encouraging them to look beyond the clinical setting. However, beginning in the third iteration, we did require a certain number of hours to be completed within local organizations through the UTMB Office of Community Engagement & Education (CEE). The service-learning initiative integrated into the community service component was the “Hospital to Home” (H2H) program. In Spring 2022, we piloted the H2H program developed by the UTMB Office of CEE in collaboration with the free, student-run clinic (St. Vincent’s Clinic) and the UTMB health system enterprise. In the next academic year, we made the H2H a required part of the community service component. In this H2H experience, medical students gain a deeper knowledge of the importance of integrating the greater social and patient context when developing care plans by identifying and addressing the unmet social needs of hospitalized patients at the bedside for smoother transitions of care. The system-level goals of this program include improving health outcomes and reducing patient readmissions.

The quarterly assignments consisted of an assortment of course activities to cover the breadth of HSS-related topics, including online modules, SDOH activity, self-reflection, essays, seminars, and Selectives depending on the iteration. We incorporated Selectives for students to have the opportunity to choose among HSS-related course activities based on their interests. The student assessment varied for each iteration; in the last two iterations, we administered the National Board of Medical Examiners (NBME) HSS Subject Examination.

### Assessment

For each cohort, we conducted mid-course and end-of-year evaluations via a curriculum management system. All course evaluations at the institution included standard questions divided into categories of Course Communication, Course Content and Materials, Assessment of Learning, Professionalism, and Overall Rating of the Course. For unique courses like HSS, the evaluations included course-specific questions that could be focused on curricular content changes. These questions were grouped into categories that varied based on the annual needs of the course, including SDOH Activity, H2H Program, Seminars, and Selectives. The evaluations included Likert scale items, single-choice responses, and open-ended responses. The evaluation included items on students’ perception of the course assignments and community service hours expected to be completed. To allow for more continuous improvement, we included two additional feedback surveys and conducted a focus group interview during the fourth iteration. In addition to the evaluations, the NBME HSS Subject Examination was added during the third iteration to assess students’ knowledge. This assessment continued for the fourth iteration.

### Analysis

For this article, we provided data for the specific items that were the focus of the iterative course changes from the course evaluations and the two feedback surveys and focus groups conducted with the fourth cohort. In addition, we provided descriptive statistics for the NBME HSS examination. Course evaluation data for the course iterations focused on the amount of material students were expected to master, SDOH Activity, H2H Program, Seminars and Selectives. Each section included Likert-scale, single or multiple response, and open-ended questions. An additional file includes the relevant items in the course evaluations and summarizes the iterative changes made to these items [see Additional File 1]. Similarly, the feedback surveys included 5 Likert-scale questions to gage students’ perceptions of the effectiveness of course communications, the usefulness of sessions, and the reasonability of course requirement; 1 multiple choice response to identify which Selectives students had completed; and 2 open-ended questions to provide feedback on strengths and areas of concern regarding the course.

The course evaluation and feedback survey data were analyzed by a team of two educators trained in data analysis, including qualitative analysis. Descriptive statistics were run for each Likert-scale question as well as for each overall category. Simple counts were used for the single or multiple response questions. The responses for the qualitative items were analyzed using an a priori category analysis. A prior category analysis was utilized since pre-determined categories were provided by evaluation. The category analysis was coupled with a strength and weakness analysis. This analysis allowed for each question and response to be examined and for themes within the categories to be developed to determine areas of strength and areas for improvement for the course.

For the focus group, general questions were asked to establish rapport, to explain the focus group process, and to allow participants to ask any questions about the process or purpose of the focus group. The specific questions related to the course iterations included:

1. What are the greatest strengths of HSS?
2. What are the most important areas for the course directors to focus on for improvement?
3. Is it reasonable to complete HSS within the variable timeframes of on- and off-cycle students?
4. What content would you recommend be moved earlier in the curriculum?
5. What other information would you like to share with the course directors about the course content, materials, or organization?

The data from the focus group was transcribed with all identifiers removed. The data was then independently analyzed by two educators trained in qualitative analysis utilizing a constant comparative analysis to determine themes. The two educators then met, discussed their findings, and agreed upon common themes, which were reported to the course directors.

## Results

**Fig. 1:**
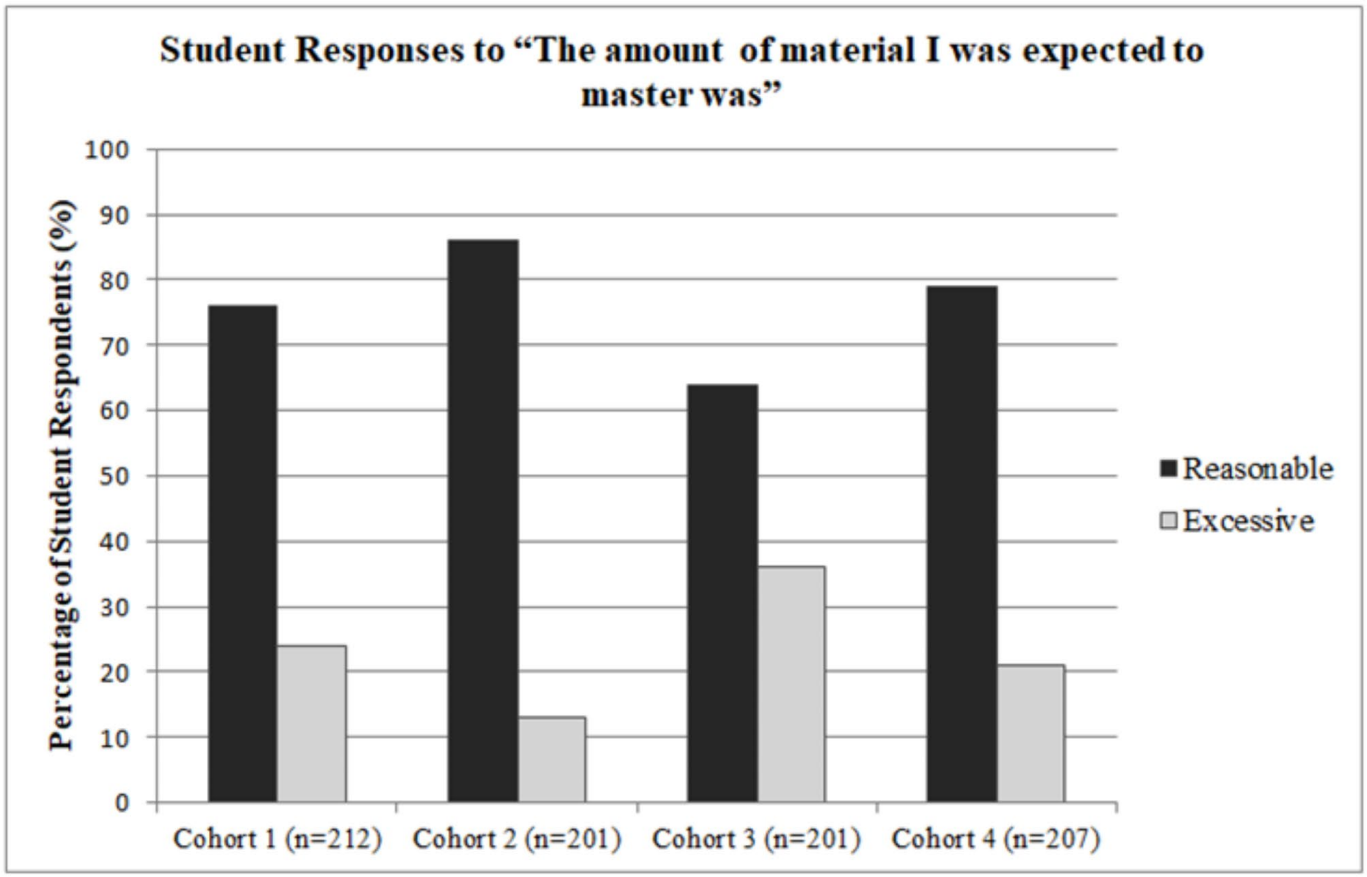
This figure shows the percentage of student responses for the question item on the amount of course material expected to master for four cohorts, 2020–2024

**Fig. 2:**
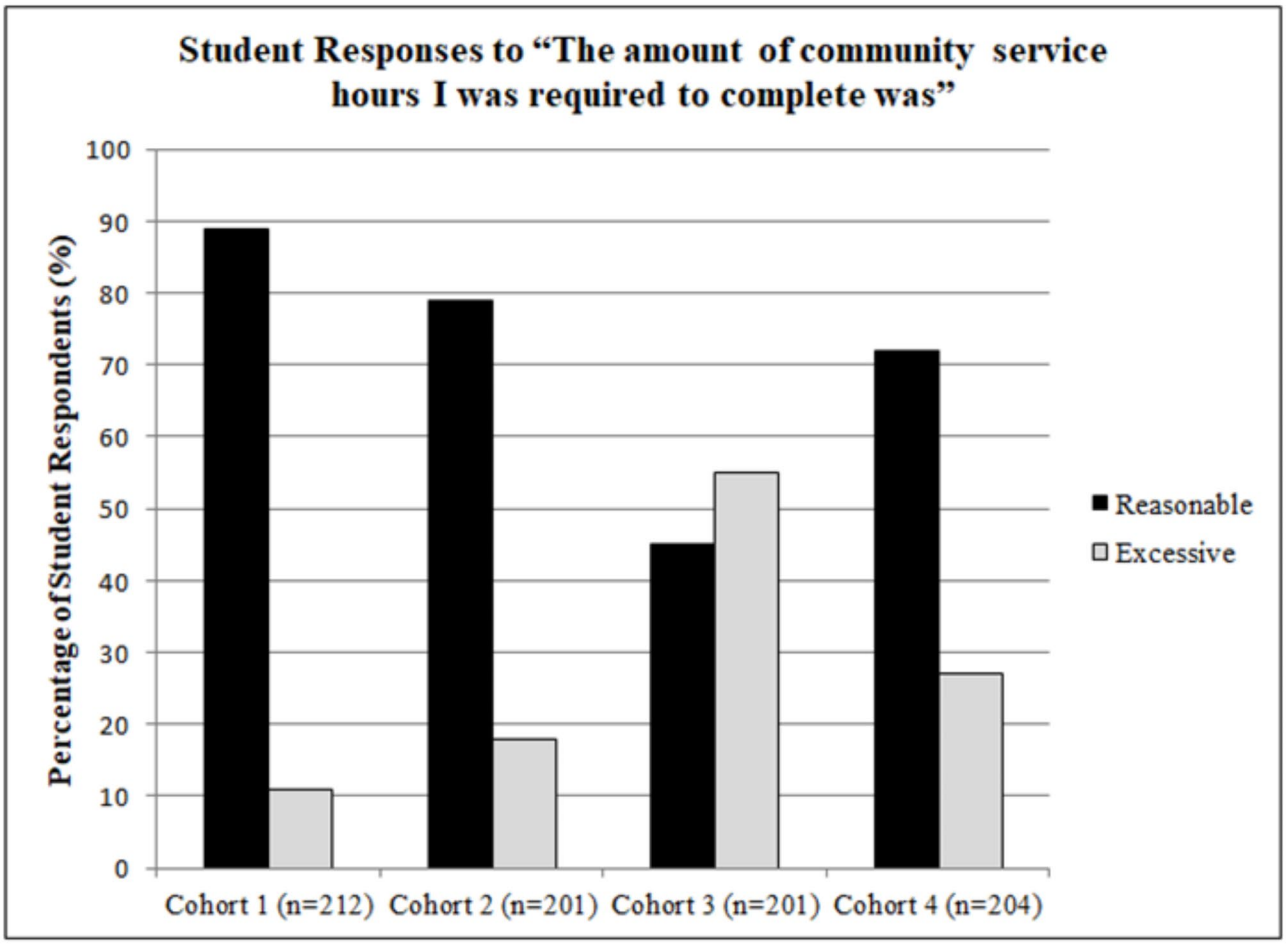
This figure displays the percentage of student responses for the question item on the amount of community service expected to complete for the course across four cohorts, 2020–2024

The response rates for the end-of-year course evaluations were 91% for cohort 1 (212/234), cohort 2 (201/221), and cohort 3 (201/220), and 94% for cohort 4 (204/217). Figure 1 shows the student responses for the amount of material the students were expected to master on a single-item question with response choices of reasonable, excessive, or not enough. The majority of students responded with “reasonable” across all four cohorts at 76%, 86%, 64% and 79%, respectively. Figure 2 summarizes the student responses for the number of community service hours in the course with the same single-item responses of reasonable, excessive, or not enough. The first two cohorts of students responded that the amount of community service hours was reasonable at 89% and 79%, respectively. With the third cohort, the majority selected excessive at 55%. Between cohorts 2 and 3, the required hours increased from 30 to 40. In the fourth cohort, 72% of the students reported that the number of hours was reasonable.

Results from the NBME exam reveal that 99.1% of examinees passed during the third and fourth iterations (*n* = 218 for cohort 3 and *n* = 216 for cohort 4). For both years, student performance on the HSS focused NBME exam was comparable to the national performance.

The themes related to the areas of strength and areas of weakness based on student comments from evaluations are highlighted in Table 1. Common themes evident across all iterations in evaluations, feedback surveys, and focus groups for areas of strength included the importance of the content, particularly for the NBME Step 2 exam and residency interviews, and the flexibility and self-paced nature of the course. One student commented, *“Great course with a lot of important information that broadened my perspective on health care systems and practice*,*”* and another student mentioned, *“Appreciated that it was self-paced and always had remote options.”* For the service-learning H2H requirement, a student responded, *“Personally*,* I found H2H to be a valuable learning experience - as medical students*,* we do not have much real-life exposure to social determinants of health and how those affect the lives of real people. I appreciated the ability to work with a social worker and other facilitators to learn more about community resources (and lack thereof). I felt like I was making a real difference in the lives of patients by providing them with resources that they otherwise would struggle to receive*,* and I appreciated the transition of care aspect as well. Transitions of care from the inpatient to the outpatient setting are so important*,* and I am grateful for the opportunity to learn more about what can hinder this and what can improve this.*” A common theme for areas of improvement in all iterations was that students felt that the online modules were redundant, and the course requirements were thought to be busy work. Another common theme was that students preferred content be taught earlier in training. One student’s comment highlights this sentiment: *“I wish medical students were taught this information more thoroughly during the MS1 year so that*,* by the time we are MS3 and MS4s*,* we could learn how to and practice integrating the principles taught in this course with direct patient care.”*

**Table 1:** This table summarizes the common themes identified for areas of strength and areas of improvement in student responses across the four cohorts, 2020–2024

Common Themes from Student Responses across the Four Cohorts, 2020–2024	
**Areas of Strengths** • Students found the content important for Step 2 exam and residency interviews. • Students appreciate the flexibility and self-paced nature of the course. • Students valued the Hospital to Home program. • Students appreciate the timely and clear course communication.	**Areas of Improvement** • Students found the course content from online modules redundant. • Students prefer the content being taught earlier in training- as it would be useful for Step exam prepping, clinical rotations, and residency process. • Students found assignments, including community service, to be too time-consuming and busy work, • Students see the value in community service, but they feel that making it required takes away from its overall purpose.

An area of strength described by cohort 2 was that the course provided students with an opportunity to interact with and give back to the community. They also found that the SDOH activities were beneficial to learning. The following year, a theme from cohort 3 was that students found the community service requirements to be too time-consuming and busy work, along with SDOH activity being redundant. For cohort 4, a general theme was that H2H sessions were seen as a valuable way to expose students to how SDOH and community resources impact care. While student comments acknowledged the value of community service across multiple cohorts, they expressed that it lost its overall purpose when it was required.

## Iterative changes based on evaluations

Table 2 provides a summary of the incremental changes across the four-course iterations. The first course iteration occurred from June 2020 to April 2021. The required components of the course in this iteration included online modules with quarterly tests, community service, writing assignments, and SDOH activity. For the online modules, the platforms used included the Institute of Healthcare Improvement (IHI) [18], the American Medical Association (AMA) Health Systems Science Learning Series [19] and Aquifer™ [20]. We piloted the community service component with a 10-hour requirement. As this was during the early part of the COVID-19 pandemic, we followed pandemic restrictions. We allowed students to select where they would complete the community service requirement. The writing assignments included an essay focusing on SDOH and a reflection on what was learned in the past year related to HSS and community service experience. The essay on SDOH used Voice Thread™ platform (https://voicethread.com/) to incorporate a peer-to-peer review component. For the SDOH activity requirement, students conducted SDOH screening for patients during their clinical rotations. After the screening, students completed a written reflection on the patients’ unmet needs and barriers and what course of action could be taken to address these. As part of assignments, we gave options to students on completing part of the requirement including piloting internally development health equity modules called “Empowering Anti-Racist Physicians in Medicine.” In the next iteration, we reframed this as Selectives to give students the opportunity to explore the HSS-related topics in which they were interested.

**Table 2:** This table highlights the incremental changes made across the four course iterations from 2020 to 2024 by course components. *COVID pandemic started

*Incremental Changes across the Four Course Iterations by Course Components*,* 2020–2024*				
	Year 1* 2020–2021	Year 2 2021–2022	Year 3 2022–2023	Year 4 2023–2024
**Community Service Component** **(Number of hours)**	10 h with community service reflection	30 h with community service reflection	40 h includes service-learning and reflection	40 h includes service-learning and reflection
**Service-Learning- Hospital to Home Program (H2H Program) (Brief Description of Changes)**	N/A	Piloted H2H Program Optional	H2H Program Required	H2H Program Required
**Quarterly Assignments** **(Brief Description of Changes)**	IHI modules, AMA HSS modules, Aquifer cases, SDOH activities, Peer reviewed essays, Reflective assignment	-Reduced online modules to IHI Basic Quality & Safety Certificate, -Added health equity module and Value-based healthcare modules & HSS seminar, -Continued SDOH activities, peer-reviewed essay, reflective assignment	- Continued IHI Basic Quality & Safety Certificate, health equity module, SDOH activities, reflective assignments, peer-reviewed essay, HSS seminars -Removed Value-based healthcare modules	-Added required orientation -Continued IHI modules for Basic Quality & Patient Safety Certificate -Shifted reflective assignments and HSS seminars to Selectives -Removed SDOH activities -Shifted health equity module into Selectives small group option -Shifted essay to Selectives small group option
**Selectives** **(Brief Description of Changes)**	Online modules, including Aquifer, piloted internally develop health equity online modules, additional community service hours	-Added quarter wrap-up sessions, HSS seminars -Continued online modules, additional community service hours	-Continued quarter wrap-up sessions, HSS seminars, online modules, additional community service hours	-Added small group options, lifestyle coaching, reflections -Increased number of HSS seminars -Continued quarter wrap-up sessions, online modules, additional community service hours
**Assessment (Brief Description of Changes)**	Quarterly Tests	Reflective assignments	NBME HSS Subject Exam	NBME HSS Subject Exam

The second iteration ran from June 2021 to April 2022. We increased the community service requirement to 30 h and continued allowing students to choose where they complete these hours, with guidance provided as needed. In Spring 2022, we piloted the H2H program. During the pilot, we allowed students to participate on a voluntary basis and have the hours count towards the community service requirement.

Based on student feedback from the first iteration of redundancy in the online content, we reduced and adjusted the required online modules for the second iteration. For the IHI modules, we reduced the required content to those modules necessary to complete the IHI Basic Quality & Safety Certificate [18]. We replaced the AMA HSS modules with the Value-Based Health Care modules by Dell Medical School and required the health equity modules mentioned that were piloted in the first iteration [21]. We received additional student feedback that quarterly tests were not helpful; these were replaced with reflections. With student feedback, we also rolled out pre-recorded didactics and live, online quarterly wrap-up sessions to help consolidate the content. We introduced other course activities, including an HSS seminar series. We changed the essay to “Essay on Patient Care” to further broaden the scope for HSS and, based on feedback, switched the platform from Voice Thread™ to Peerceptiv™ (https://peerceptiv.com). The SDOH activities requirement was maintained.

In the third course iteration, conducted from June 2022 to April 2023, we made major changes based on multiple factors, including a successful pilot of the H2H program, approval for HSS NBME administration, and student feedback. Community service was expanded with a service-learning component which required students to complete two H2H sessions included in their 40-hour community service requirement, along with completing part of their community service hours through the opportunities set by the Office of CEE partnered with local organizations. Simultaneously, the H2H program was also offered to first-year medical students, meeting the recommendation to integrate content earlier in the curriculum. The fourth-year students in our course had the opportunity to sign up for sessions and be paired with first-year medical students in which they took a mentoring role in this service-learning experience.

Due to continued redundancy in content and technical issues with the Value-Based Health Care modules by Dell, we reduced the module requirement to only the IHI Basic Quality & Safety Certificate and the internally developed health equity modules. We continued the pre-recorded didactics, quarter wrap-up sessions, self-reflections, Peerceptiv™ assignment, and Selectives. The seminar series was further expanded. For the student assessment component in this iteration, we added the HSS NBME Subject Exam.

For the fourth course iteration in June 2023 to April 2024, we reduced the requirements and increased Selectives based on student feedback regarding the complexity of the course. This process included removing writing assignments as requirements and shifting reflections to count towards Selectives. In the third iteration, SDOH activities were kept as a means of reinforcing learning from H2H; however, since students found this repetitive, by the fourth iteration, we no longer required it. We continued to require the IHI online modules. While we retained the community service requirements, including the number of hours and the H2H program, we no longer required students to complete part of their community service through the Office of CEE. The community service through the Office of CEE became optional based on student feedback and the flexibility needed for student schedules, including away rotations and residency interviews. In addition, student feedback indicated a need to provide more clarification on expectations. Whereas prior iterations had recorded orientation available, at this iteration we required students to attend live orientation to review course expectations and clarify key concepts like the difference between community service, service-learning, and volunteering. In this iteration, students were no longer required to attend Seminars, although we still offered the series of sessions which counted towards Selectives. We expanded the Selectives options to include small group activities. We transitioned the required health equity online modules into a Selectives option for small group activity called “Anti-Racism in Medicine Journal Club.” The other small group was “Patient and Health Care Case Discussions,” which was transformed from the previously required Essay on Patient Care.

## Discussion

A key strategy for implementing this course was taking an iterative approach by setting a foundation with conceptual- and experiential-based learning, then building up and expanding these components each year. For instance, we piloted the H2H program in the second course iteration with a small number of students and then, based on students’ feedback, scaled it up as a requirement the following year after ensuring administrative and logistical readiness. Such an adaptive approach allowed for the fine-tuning of existing curricula and vetting of newly created curricular elements.

As the curriculum evolved in stages, we were interested in student perception of the quantity of course material and community service requirements. In the first two cohorts, students indicated that the course material was manageable. In the third cohort, fewer students, but still the majority, felt the workload was reasonable. By the fourth cohort, the percentage of students who found the workload reasonable had increased. In terms of community service hours, the first two cohorts felt the amount was reasonable. However, the third cohort viewed the hours as excessive. This changed again, with students in the fourth cohort agreeing that the number of hours was reasonable. A possible contributing factor to the slightly negative responses of the third cohort was likely multiple changes to the course, including the service-learning requirement (H2H program), which created more structured mandatory activities along with the addition of the NBME exam. Another potential reason for the higher number of negative responses in the third cohort could be that this group included more students who were resistant to a course still in its early stages and introducing new service-learning experiences.

Our study uncovered a recurring theme in which students recognized the course content as valuable, but felt it was overly time-consuming and consisted of unnecessary tasks. The literature describes HSS as the “broccoli” of medical education because students understand its importance but struggle to engage fully with the material because of competing priorities [6]. Our course encountered similar challenges and tackled student engagement through an iterative approach and in accordance with the best practices of medical education. For example, it was clear from the feedback of the third cohort’s students that we need to revise the course content and distinguish between community service, service-learning, and volunteering. Community service, service-learning, and volunteering are often associated with mixed perceptions due to varying interpretations of their purpose, structure, and impact [9, 22, 23].

Implementation of this course involved several limitations worth noting. One significant limitation relates to the reliance on self-reported data, which may introduce biases that can skew the results. Additionally, although the course demonstrated efficacy in enhancing knowledge, primarily evidenced by performance on the NBME exam, its evaluation primarily centers on knowledge-based assessments pertaining to lower-order Bloom’s taxonomy. This focus may inadvertently sideline crucial aspects of skill acquisition and application, thereby potentially reinforcing the hidden curriculum in medical education. Finally, educators are most eager to learn the long-term impact of this course on students’ professional development and their patient outcomes. Our course is in the early implementation stage, and as it progresses, we are interested in obtaining alumni feedback. Understanding how the skills acquired in the course translate into tangible benefits for patients is crucial for refining the curriculum and assessing its actual effectiveness in meeting healthcare needs.

As we navigate the next iteration, the course has undergone improvements to better meet student needs. Based on student feedback, we expanded the seminar series and more intentionally included how the course objectives, online modules, and community service component align and overlap for the various sessions offered. Concurrently, there is recognition of the importance of introducing HSS concepts and experiences earlier in medical school. While the H2H program had already been woven into the first-year curriculum, paving the way for earlier exposure and continuity, we are collaborating with key partners to determine the next steps for integrating HSS content sooner. Future iterations of this post-clerkship course will focus on advanced content and skills development, aligning with our commitment to offer comprehensive HSS education.

## Conclusion

As more medical schools revamp their curriculum to include health systems science, they will need guidance, including real-world experience, on how to integrate this third pillar of medical education. We describe the implementation of a post-clerkship course on health systems science, which integrates it with community service and service-learning. A major lesson learned is the need to take an iterative approach in building components like service-learning and addressing challenges, including student buy-in.

## Electronic supplementary material

Below is the link to the electronic supplementary material.

Additional File 1: Course Evaluation Question Iterations: A table providing a summary of the iterative changes to course evaluation questions from 2020 to 2024. All questions are on a 5-point Likert Scale unless otherwise noted

## Data Availability

The datasets used and/or analysed during the current study are available from the corresponding author on reasonable request.
