# Supplementary material for: Lessons learned from implementing health systems science and community service course for fourth-year medical students
Source: BMC Med Educ. 2025 Apr 15;25:542. doi: 10.1186/s12909-025-07137-3 (PMC12001676; doi:10.1186/s12909-025-07137-3)
Supplement: Supplementary file 1 — Additional File 1: Course Evaluation Question Iterations: A table providing a summary of the iterative changes to course evaluation questions from 2020 to 2024. All questions are on a 5-point Likert Scale unless otherwise noted [file 12909_2025_7137_MOESM1_ESM.pdf]

Additional File 1

Course Evaluation Question Iterations

The following table provides a summary of the iterative changes of course evaluation questions from 2020 to 2024. All questions are on a 5-point Likert Scale unless otherwise noted.

	2020-2021	2021-2022	2022-2023	2023-2024
**Course Communications**				
The following information was provided in a timely manner: schedules, events, deadlines	X	X	X	X
course objectives	X	X	X	X
learner responsibilities	X	X	X	X
assessment methods	X	X	X	X
grading criteria	X	X	X	X
The course director(s) was/were available to answer my questions in a timely manner.	X	X	X	X
The course coordinator(s) (staff) was/were available to answer my questions in a timely manner.	X	X	X	X
Please leave any comments about course communications below. (open-ended)	X	X	X	X
**Course Personnel**				
The course director (physician) was supportive of my learning.	X	X	X	Removed
The course coordinator (staff member) was supportive of my learning.	X	X	X	Removed
**Course Content and Materials**				
The content in this course focused on health systems science.	X	X	X	X
The required online modules in this course were important to my mastery of the content.	X	X	X	X
The required online modules in this course were aligned with learning objectives.	X	X	X	X
The online modules in this course challenged me to fill gaps in my knowledge.		X	X	X
The online modules in this course challenged me to think critically.		X	X	X
The online modules in this course challenged me to integrate health systems science with clinical and basic sciences.		X	X	X
The supplemental resources provided in this course were important to my mastery of the content.		X	X	X
The content and materials were structured in a clear manner online.	X	X	X	X
The amount of material I was expected to master was: Excessive, Reasonable, Not Enough (single choice)	X	X	X	X
Please list any outside resources you found useful in mastering the course content. (open-ended)	X	X	X	X

Please leave any comments about Course Content below. (open-ended)	X	X	X	X
**Pre-Recorded Didactic Sessions**				
The pre-recorded didactic sessions provided in this course were important to my mastery of the content.		X	X	
The pre-recorded didactic sessions provided in this course were aligned with the learning objectives.		X	X	
The pre-recorded didactic sessions provided in this course filled gaps in my knowledge.		X	X	
The pre-recorded didactic sessions provided in this course challenged me to think critically.		X	X	
The pre-recorded didactic sessions provided in this course integrate health systems science with clinical and basic sciences.		X	X	
Please use this space to leave any additional comments about pre-recorded didactic sessions. (open-ended)		X	X	
**Quarter Wrap Up Sessions**				
The Quarter Wrap-Up sessions provided in this course were important to my mastery of the content.		X	X	
The Quarter Wrap-Up sessions provided in this course were aligned with the learning objectives.		X	X	
The Quarter Wrap-Up sessions provided in this course filled gaps in my knowledge.		X	X	
The Quarter Wrap-Up sessions provided in this course challenged me to think critically.		X	X	
The Quarter Wrap-Up sessions provided in this course integrate health systems science with clinical and basic		X	X	
Please use this space to leave any additional comments about Quarter Wrap-Up sessions. (open-ended)		X	X	
**Seminar Sessions**				
The Seminar sessions provided in Quarter 3 this course were important to my mastery of the content.		X	X	
The Seminar sessions provided in Quarter 3 were aligned with the learning objectives.		X	X	
The Seminar sessions provided in Quarter 3 filled gaps in my knowledge.		X	X	
The Seminar sessions provided in Quarter 3 challenged me to think critically.		X	X	
The Seminar sessions provided in Quarter 3 integrate health systems science with clinical and basic sciences.		X	X	
Please use this space to leave any additional comments about Seminar sessions. (open-ended)		X	X	
**Selective Activities / Instructional Methods**				
Which of the following Selective options have you completed to date? Please select all that apply.				X
If you chose Miscellaneous from the choices above, please write in a description below. (open-ended)				X
The Selective Activities provided in this course were important to my mastery of the content.	X	X	X	X

The Selective Activities provided in this course were aligned with the learning objectives.	X	X	X	X
The Selective Activities provided in this course filled gaps in my knowledge.	X	X	X	X
The Selective Activities provided in this course challenged me to think critically.		X	X	X
The Selective Activities provided in this course integrate health systems science with clinical and basic sciences. /The online modules and assignments/activities in this course challenged me to integrate health systems science with clinical and basic sciences. (Wording changed)	X	X	X	X
Please leave any comments about specific Selective Activities below. (open-ended)		X	X	X
Please describe any positive experience from the online experience. (open-ended)	X			
Please describe any recommendations for areas of improvement for the online experience. (open-ended)	X			
**Social Determinates of Health**				
The Social Determinants of Health (SDOH) Activities gave me the opportunity to interview and screen patients for social risk factors that affect their health.	X	X	X	
The online modules with content on SDOH provided me with sufficient knowledge to perform the SDOH activities.	X	X		
The SDOH activities enhanced my abilities to recognize the barriers to health and gaps of care that patients of diverse socioeconomic backgrounds potentially face.	X	X	X	
The SDOH activities allowed me to apply and integrate my knowledge of social determinants to determine next steps to address barriers to patient care.	X	X	X	
The SDOH activities increased my appreciation of the importance of recognizing and addressing the social determinants of health as part of patient care.	X	X	X	
Please use this space to leave any additional comments about the SDOH activities here. (open-ended)	X	X	X	
I had sufficient prior knowledge on SDOH from prior courses to perform the H2H and SDOH activities.			X	
**Service Learning**				
The H2H activity was well organized.			X	X
I was given sufficient preparation for the H2H activity.			X	X
The small group debriefing session at the end was effective.			X	X
I would recommend participating in the H2H activity to other students.			X	X
After the H2H activity, I will change the practice of taking care of patients.			X	X
I would voluntarily participate in this program again.			X	X
H2H activities enhanced my abilities to recognize the			X	X

barriers to health and gaps of care that patients of diverse socioeconomic backgrounds potentially face.				
The H2H activities allowed me to apply an integrate my knowledge of social determinants to determine the next steps to address barriers to patient care.			X	X
The H2H activities increased my appreciation of the importance of recognizing and addressing the social determinants of health as part of patient care.			X	X
**Community Service**				
The community service component increased my knowledge on community resources available.				X
The community service component gave me a better understanding of the broader meaning of health and taking care of patients in the greater social context.				X
The amount of community service hours I was required to complete was: Excessive, Reasonable, Not Enough	X	X	X	X
Please leave any comments about Service Learning below. (open-ended)				X
Please describe the strengths of the community service/service-learning component of the HSS course. (open-ended)	X	X	X	
Please describe areas of improvement of the community service/service-learning component of the HSS course. (open-ended)	X	X	X	
**Assessment of Learning**				
The course assignments were aligned with the course learning objectives.		X	X	X
The course assignments aligned with the course content.		X	X	X
The course assignments challenged me to fill gaps in my knowledge.		X	X	X
The course assignments afforded me the opportunity to think critically.		X	X	X
The course assignments integrated health systems science with clinical and basic sciences.		X	X	X
The course assignments were important to my mastery of the content.		X	X	X
The high stakes exam (NBME) aligned with specific course objectives.				X
The high stakes exam (NBME) aligned with the course content.				X
I received (formal) formative feedback midway through the course.	X	X	X	X
The assignments and exams: aligned with course objectives	X			
The assignments and exams: aligned with the course content	X			
Please leave any comments about the Assessment of Learning below. (open-ended)	X	X	X	X
**Professionalism**				

Professional attitudes and behaviors were demonstrated and promoted by course coordinator(s).	X	X	X	X
Professional attitudes and behaviors were demonstrated and promoted by course director(s).	X	X	X	X
Professional attitudes and behaviors were demonstrated and promoted by peers.	X	X	X	X
I was provided with information about how to report student mistreatment during this course.		X	X	X
Please leave any comments about the Assessment of Learning below. (open-ended)	X	X	X	X
Please leave any comments about the Assessment of Learning below. (open-ended)	X	X	X	X
**Overall Rating of Course**				
Overall, how satisfied were you with this course?				X
To what extent did this course support you in attaining your learning goals?				X
Overall rating of course			X	
**Online Experience**				
Please describe any positive experience from the online experience. (open-ended)		X	X	X
Please describe any recommendations for areas of improvement for the online experience. (open-ended)		X	X	X
**General Comments**				
Please comment on specific areas of strength in this course. (open-ended)	X	X	X	X
Please comment on specific areas for improvement in this course. (open-ended)	X	X	X	X
Please leave any additional comments about your course experience below. (open-ended)	X	X	X	X
